# A study on the development of a fitness age prediction model: the national fitness award cohort study 2017–2021

**DOI:** 10.1186/s12889-024-19922-8

**Published:** 2024-09-27

**Authors:** Dong Hyun Yoon, Jeong-Hyun Kim, Shi-Uk Lee

**Affiliations:** 1 Department of Rehabilitation Medicine, Seoul National University College of Medicine, Seoul, Republic of Korea; 2 Department of Rehabilitation Medicine, Seoul Metropolitan Government Boramae Medical Center, Seoul, Republic of Korea; 3 Institute on Aging, Seoul National University, Seoul, Republic of Korea; 4 Department of Physical Medicine & Rehabilitation, Seoul National University College of Medicine, Seoul National University Boramae Medical Center, 20, Boramae-ro 5-gil, Dongjak-gu, Seoul, 07061 Korea

**Keywords:** Fitness age, Aging, Fitness assessment, Health outcomes

## Abstract

**Background:**

Physical fitness is considered an important indicator of the health of the general public. In particular, the physical fitness of the older adults is an important requirement for determining the possibility of independent living. Therefore, the purpose of this study was to examine the association between chronological age and physical fitness variables in the National Fitness Award Cohort study data and to develop multiple linear regression analyses to predict fitness age using dependent variables.

**Methods:**

Data from 501,774 (359,303 adults, 142,471 older adults) individuals who participated in the Korea National Fitness Award Cohort Study from 2017 to 2021 were used. The physical fitness tests consisted of 5 candidate markers for adults and 6 candidate markers for the older adults to measure muscle strength, muscle endurance, cardiopulmonary endurance, flexibility, balance, and agility. Pearson’s correlation and stepwise regression analyses were used to analyze the data.

**Results:**

We obtained a predicted individual fitness age values from physical fitness indicators for adults and older adults individuals, and the mean explanatory power of the fitness age for adults was [100.882 – (0.029 × VO_2_max) – (1.171 × Relative Grip Strength) – (0.032 × Sit-up) + (0.032 × Sit and reach) + (0.769 × Sex _male = 1; female = 2_)] was 93.6% (adjusted R^2^); additionally, the fitness age for older adults individuals was [79.807 – (0.017 × 2-min step test) – (0.203 × Grip Strength) – (0.031 × 30-s chair stand) – (0.052 × Sit and reach) + (0.985 × TUG) – (3.468 × Sex _male = 1; female = 2_) was 24.3% (adjusted R^2^).

**Conclusions:**

We suggest the use of fitness age as a valid indicator of fitness in adults and older adults as well as a useful motivational tool for undertaking exercise prescription programs along with exercise recommendations at the national level.

## Introduction

Due to the increase in life expectancy, the era of the 100-year-old generation is expected to occur at a rapid pace [1, 2]. According to previous studies, chronic diseases such as high blood pressure, diabetes, and high cholesterol are generally increasing due to the increased prevalence of obesity in adults over the age of 30 [3–5]. However, despite the spread of chronic diseases, efforts to prevent them are insufficient.

The physical fitness level is considered an important indicator of the health of the general public [6–9]. In particular, the physical fitness of the older adults is an important criterion for determining the possibility of independent living [10, 11]. Physical fitness includes factors related to health conditions such as musculoskeletal and cardiorespiratory fitness [9, 12]. Typical physical fitness tests include muscle strength (hand grip strength tests) [6, 7, 13], muscular endurance (30-s chair stand test and sit-ups test) [6, 13, 14], flexibility (chair sit-and-reach test) [13, 15], balance (time up-and-go) [7], and cardiopulmonary endurance (graded exercise test and 2-minute step test) [16–18]. The association between physical fitness and health status has been reported in several studies [8, 18–20]. Therefore, due to the influence of lifestyle, environment, and epigenetics, each individual experiences the physical aging process differently, and which is why many studies have focused on biological age [19, 21, 22]. Biological age gradually decreases in viability over time and increases with age, which ultimately leads to disability and mortality [21]. The concept of physical fitness age is not simply measuring the passage of time, but a major determinant of health status, measuring the progression of changes in physical function in the aging process, and the need for physical fitness age in aging studies is increasing. Although a few studies emphasized the importance of physical fitness age [2, 7, 13], large-scale and simplified reports on fitness age estimation formulas are needed. To date, the studies reported have limitations on small sample sizes and limited age groups, while complexities of measurement variables used further impede the development of simplified estimation formulas [23, 24].

Therefore, the aim of this study was to examine the association between chronological age and physical fitness variables in the National Fitness Award Cohort study data, which collects more than 100,000 data each year for Koreans. We aimed to develop multiple linear regression analyses to predict fitness age using dependent variables (e.g., muscle strength, muscular endurance, cardiopulmonary endurance, flexibility, balance). To the best of our knowledge, this study is the first nationwide to develop simplified estimation formulas for the “fitness age.” It is expected that this study will present major trends and standardizations in future health-related fitness age while enabling customized services for exercise by providing the foundation for precise frailty classification, especially in older adults.

## Methods

### Data sets and study participants

A total of 501,774 (male = 236,852 and female = 264,922) participants in the National Physical Fitness 100 Health Promotion Class exercise program at 82 sites nationwide (76 sites, 6 mobile sites) in South Korea from 2017 to 2021. The criteria for selecting subjects were adults and the older adults who voluntarily agreed after fully explaining the purpose and content of the study. Participants who’s physical strength could not measure their physical strength due to acute cardiovascular disease, systemic infection, and musculoskeletal damage were excluded from the study. The physical characteristics of the study subjects are shown in < Table 1>. Ethical approval of this retrospective study was given by the Institutional Review Board (IRB) of the Seoul National University Boramae Medical Center, and all subjects provided their written informed (IRB No. 07-2024-2).

**Table 1:** Baseline characteristics of subjects

	Adults			older adults		
	Male *n* = 182,972	Female *n* = 176,331	Total *n* = 359,303	Male *n* = 53,880	Female *n* = 88,591	Total *n* = 142,471
Age, yrs	35.58 ± 13.82	42.66 ± 14.49	39.06 ± 14.59	73.26 ± 5.28	72.41 ± 5.51	72.73 ± 5.44
Height, cm	173.05 ± 6.19	159.26 ± 5.83	166.28 ± 9.15	165.24 ± 5.86	152.48 ± 5.49	157.29 ± 8.36
Body weight, kg	74.44 ± 10.87	58.25 ± 8.62	66.49 ± 12.74	66.69 ± 8.88	57.62 ± 7.96	61.04 ± 9.41
Body fat, %	21.79 ± 6.67	31.05 ± 6.80	26.33 ± 8.17	26.38 ± 6.71	35.16 ± 14.43	31.85 ± 12.84
BMI, kg/m^2^	24.75 ± 2.97	22.92 ± 3.19	23.85 ± 3.21	24.45 ± 10.13	24.82 ± 9.17	24.68 ± 9.54
Waist cir, cm	85.45 ± 9.10	79.24 ± 8.67	82.10 ± 9.40	86.07 ± 8.45	83.08 ± 8.30	84.24 ± 8.48
Grip Strength, kg	34.35 ± 10.56	30.50 ± 10.38	32.46 ± 10.65	31.19 ± 6.57	19.65 ± 4.79	24.00 ± 7.87
Relative HGS, %	54.91 ± 11.79	49.60 ± 12.04	52.31 ± 12.20	50.67 ± 9.64	37.58 ± 8.51	42.52 ± 10.97
Sit and reach, cm	11.85 ± 9.22	11.99 ± 9.27	11.92 ± 9.25	4.17 ± 9.61	13.28 ± 8.42	9.85 ± 9.93
VO_2_max, ml/kg/min	38.95 ± 6.30	37.06 ± 5.97	37.96 ± 6.20			
Sit-up, n	33.92 ± 15.53	29.31 ± 15.72	31.66 ± 15.79			
2-min step test, n				109.09 ± 23.15	102.23 ± 26.27	104.85 ± 25.34
30-s chair stand, n				21.31 ± 6.68	18.57 ± 6.35	19.60 ± 6.62
TUG, sec				6.01 ± 1.68	6.77 ± 28.15	6.48 ± 22.24

BMI; body mass index, Waist cir; waist circumference, TUG; time up and go, HGS; handgrip strength, Data are reported as the mean ± standard deviation

### Body composition

Body composition was measured using multifrequency impedance (Inbody 720, Biospace Co., Seoul, Korea), and all metallic materials were removed, shoes and socks were removed, both feet were placed on the electrodes of the tread plate while wearing light clothing, and the electrodes of both handles were held correctly and the arms were spread out approximately 30°. The results were measured once, and the figures on the results were recorded. Body mass index (BMI) was calculated using the ratio of weight squared to height (kg/m^2^). The height was measured in units of 0.1 cm after the study participants did not tilt their heads sideways in a natural upright position with their backs to the renal system and standing barefoot. The waist circumference was relaxed and straightened as much as possible and then measured horizontally, and the navel area was measured [2, 25].

### Muscle strength (grip strength test)

Handgrip strength was measured, and in the upright position, the grip of the grip meter (GRIP-D 5101; TAKEI, Co., Japan) was held at the middle phalanx of the finger. The arm was straightened and the body and arm were pulled at 15 degrees for 5 s. The highest value was recorded in units of 0.1 kg by conducting two times on the left and right sides. Relative grip strength (%) was defined by the following formula: absolute handgrip strength (kg)/body weight (kg) × 100 [6, 25].

### Muscular endurance (30-s chair stand test and sit-ups)

Muscular endurance was measured by sitting on a chair and standing up and sip-ups. The participants sat in the center of the chair, with the soles of both feet touching the ground, and the arms crossing in front of the chest. The participants sat on the chair for 30 s with the start chant and repeatedly stood up. If the study participant stood up halfway through at the end of 30 s, this was considered the number of times he stood up. The sit-up was performed by raising both elbows to the thigh after lying on the back of the mat with the knees bent approximately 30 cm away from the hip, and raising the upper body after the signal started, and the measurement unit was recorded as the number of measurements was recorded for 1 min [6].

### Cardiopulmonary endurance (2-min step test and estimated VO_2_max)

Cardiopulmonary endurance was measured by walking in place for two minutes and estimating VO_2_max. After and after adjusting the height by attaching a rubber band to both pillars of the support at the same height as the point marked on the thigh, the study participants raised the knee from the right foot with the start signal. The number of repetitions of both feet was recorded as one and performed for 2 min. A graded exercise treadmill test using the Bruce protocol was applied to measure VO_2_max. All participants started walking at a speed of 2.7 km/h with a slope of 10%. The speed was increased by 1.3–1.4 km/h at 3-minute intervals, and the incline was increased by 2% during each stage. Graded exercise tests were performed on treadmills (TM55 treadmills; Quinton Cardiology Systems, Inc., Seattle, WA, USA). Heart rate was measured every minute using a heart rate monitor (Quinton Q-Stress, Quinton Cardiology Systems, Inc., Bothell, WA, United States) and the change in heart rate was measured on recovery for three minutes immediately after the exercise stop. Participants were expected to reach one of three of the following criteria: (1) a heart rate reserve rate > 85%, (2) a heart rate that did not increase with increasing steps, (3) a rating of perceived exercise > 17 (range: 6–20), and (4) absence of stop request [16, 17]. The VO_2_max was calculated using the Bruce formula: 6.70 − 2.82 × (1: male, 2: female) + (0.056 × exercise maintaining time (s)) (Bruce et al., 1973). The VO_2_max was calculated using the Bruce formula: 6.70 − 2.82 × (1: male, 2: female) + (0.056 × exercise maintaining time (s)) (Bruce et al., 1973).

### Flexibility (sit-and-reach)

The Participants sat on the floor, straightened their knees, brought the soles of their feet into close contact with the measuring instrument, and let both hands extend forward. The upper body was bent to extend to push the measuring plate as much as possible, and the maximum value was recorded in units of 0.1 cm after two measurements [13, 15].

### Balance (time up-and-go)

Balance was measured by sitting on a chair and returning to the 3 m target. After sitting in the center of the chair, both hands were placed on the thigh, and when the signal sounded, the cone at 3 m from the chair returned as fast as possible to measure the time it took to sit back in the chair. At this time, when the start signals sounded, measurements were made regardless of whether the study participants started moving. Measurements were made twice and the maximum value was recorded at 0.001 Sect [7].

### Statistical analyses

Statistical analyses were performed using the Statistical Package for the Social Sciences (SPSS) version 25.0 (IBM Corporation, Armonk, NY, US). The mean and standard deviation were calculated for all the measured parameters. Descriptive statistical analysis, Pearson’s correlation analysis, and stepwise regression analysis were also conducted. Pearson’s correlation analysis was used to analyze the relationships between age and physical fitness parameters in adult and older adults. Stepwise regression analysis was applied to develop of the prediction equations according to age group. To perform multiple linear regression analysis, the β-value (the regression coefficient) was used to verify whether the independent variables had explanatory power [26]. In this work, we used the stepwise mode of regression analysis, which is indicated when multiple independent variables are taken as predictors. The threshold for statistical significance was considered with a *p*-value < 0.05.

## Results

Participants were stratified into two groups (adult and older adults). The average age of the adult participants was 39.06 years (± 14.59 SD), height was 166.28 cm (± 9.15 SD), and weight was 66.49 kg (± 12.74 SD); 49.08% of all the participants were female. The average age of the older adults was 72.73 years (± 5.44 SD), the average height was 157.29 cm (± 8.36 SD), and the average weight was 61.04 kg (± 9.41 SD); 62.32% of all the participants were female. The detailed characteristics of the participants are shown in Table 1.

To develop the fitness age estimation formulas for Korean adults and the older adults, this study conducted stepwise regression analysis with health-related fitness components as independent variables and age as the dependent variable. Table 2 shows the results of the regression analysis using physical fitness parameters without outlier data. The explanatory power of the fitness age regression models (*n* = 359,303) for adults was 93.6%, and the SEE was 3.634 (t = 614.838, *P* < .001). Moreover, the explanatory power of the developed fitness age regression models (*n* = 142.471) for elderly individuals was 24.3%, and that for SEE was 4.793 (t = 397.012, *P* < .001) (Table 3). Finally, the formula for calculating fitness age is as follows:

1. Fitness age for adults: 100.882 – (0.029 × VO_2_max) – (1.171 × Relative Grip Strength) – (0.032 × Sit-up) + (0.032 × Sit and reach) + (0.769 × Sex _male = 1; female = 2_).
2. Fitness age for older adults: 79.807 – (0.017 × 2-min step test) – (0.203 × Grip Strength) – (0.031 × 30-s chair stand) – (0.052 × Sit and reach) + (0.985 × TUG) – (3.468 × Sex _male = 1; female = 2_).

**Table 2:** Regression equation predicting fitness age without outlier data in adults

		B	SE	β	*t*	*P*-value	*R*	Adjusted *R*^2^	SEE
Adults	(Constant)	100.882	0.164		614.838	0.000	0.967	0.936	3.634
	VO_2_max	− 0.029	0.005	− 0.013	-6.334	0.000			
	Relative HGS	-1.171	0.002	− 0.932	-496.486	0.000			
	Sit-up	− 0.032	0.002	0.033	-17.175	0.000			
	Sit and reach	0.032	0.002	0.020	14.911	0.000			
	Sex	0.769	0.039	0.027	19.527	0.000			

Data are presented as SEE, standard error of estimation; Sex, 1 = Male, 2 = Female

**Table 3:** Regression equation predicting fitness age without outlier data by older adults’ individuals

		B	SE	β	*t*	*P*-value	*R*	Adjusted *R*^2^	SEE
older adults	(Constant)	79.807	0.201		397.012	0.000	0.493	0.243	4.793
	2-min step test	− 0.017	0.001	− 0.064	-20.041	0.000			
	Grip Strength	− 0.203	0.003	− 0.281	-67.870	0.000			
	30-s chair stand	− 0.031	0.003	− 0.031	-9.058	0.000			
	Sit and reach	− 0.052	0.002	− 0.093	-28.053	0.000			
	TUG	0.985	0.012	0.291	82.596	0.000			
	Sex	-3.468	0.050	− 0.302	-69.217	0.000			

Data are presented as SEE, standard error of estimation; Sex, 1 = Male, 2 = Female

Table 4 shows the relationships between age and the predicted fitness age parameters in adult and older adults’ groups. The measured fitness age parameters were related to the predicted VO_2_max (*r* = − .637, *P* < .01), Relative Grip Strength (*r* = − .956, *P* < .01), Sit-up (*r* = − .616, *P* < .01), 2-min step test (*r* = − .271, *P* < .01), Grip Strength (*r* = − .202, *P* < .01), 30-s chair stand (*r* = − .309, *P* < .01), Time Up and Go (*r* = − 26, *P* < .01), sit and reach (*r* = .011, *P* < .01, *r* = − .250, *P* < .01, respectively), and Sex (*r* = .243, *P* < .01, *r* = − .076, *P* < .01, respectively).

**Table 4:** Correlation coefficients between dependent variables and fitness age parameters

		*r*	*P*-value
adults			
	VO_2_max	− 0.637	*P* < .01
	Relative Grip Strength	− 0.956	*P* < .01
	Sit-up	− 0.616	*P* < .01
	Sit and reach	0.011	*P* < .01
	Sex	0.243	*P* < .01
older adults			
	2-min step test	− 0.271	*P* < .01
	Grip Strength	− 0.202	*P* < .01
	30-s chair stand	− 0.309	*P* < .01
	Sit and reach	− 0.250	*P* < .01
	Time Up and Go	0.426	*P* < .01
	Sex	− 0.076	*P* < .01

Data are presented as Sex, 1 = Male, 2 = Female

## Discussion

In this study, we examined the association between chronological age and physical fitness variables in the National Fitness Award Cohort study data and developed multiple linear regression analysis to predict fitness age using dependent variables (e.g., muscle strength, muscular endurance, cardiopulmonary endurance, flexibility, balance, agility). Statistical analysis was conducted by removing outliers to prevent an increase in prediction error before developing a multiple linear regression model to predict fitness age. As a result, the mean explanatory power of the predicted fitness age test regression model in adults (age 20–64) was 93.6%, suggesting our prediction model is practical enough. However, the mean explanatory power for the prediction model of fitness age in older adults (age over 65) was 24.3%, indicating more research is required to develop a suitable prediction model for older adults. For example, for adults, we used VO2 max estimation formula using graded exercise treadmill test. However, for older adults aged over 65, cardiopulmonary function was evaluated using a 2-minute step test method, the fitness prediction model we have in this study need to consider this factor.

In recent decades, several studies have been reported since the concept of physical fitness age was proposed in the field of gerontology, and regarding aging and physical health, physical fitness age is a comprehensive evaluation of physical fitness rather than individual chronological age [27]. Therefore, rather aging induced physiological and psychological reduction over time, specific health related components such as musculoskeletal and cardiopulmonary are more significant factor in evaluating fitness level. Several studies have also reported that physical fitness age can be used to evaluate an individual’s physical fitness [13, 28] and that physical fitness age is a concept to make it easier to understand the physical fitness data of adults and older adults people [7]. Several studies have been conducted to estimate physical age. According to a report by Kimura et al. [13], five associated physical fitness indicators related to physical age (i.e., grip strength, vertical jump, functional reach, one leg stand with eyes open, and 10 m walking time) were determined in a seven-year longitudinal study of 122 healthy older adults. Additionally, Latorre et al. [7] obtained the functional fitness age formula for 459 older adults females. The test included six measurements: the arm curl test, back scratch test, chair stand test, chair sit-and-reach test, and 8-foot up-and-go test. However, both studies were limited in their study due to their small sample size and could not include only females as participants or include older adults risk variables such as vertical jumps and arm-curls to generalize the measurement variables they used.

To compensate for the limitations of previous studies, this study used data from the National Fitness Award Cohort study of approximately 500,000 adults from 2017 to 2021. In conclusion, we found health-related physical fitness indicators of fitness for fitness age (5 candidate markers for adults and 6 candidate markers for older adults) using step-by-step selection methods and identified fitness indicators such as muscle strength, muscle endurance, cardiopulmonary endurance, flexibility, balance, and agility as health-related physical fitness component variables. This study predicted individual fitness age values from physical fitness indices for adults and older adults individuals, and the mean explanatory power of the fitness age regression model was 93.6% (adjusted R^2^) and 24.3% (adjusted R^2^). The variables measured for each group were determined in advance by studies conducted by national institutions [29], and the researchers believe that a physical health assessment based on body age would be advantageous and useful for estimating an individual’s physical health age.

Physical activity and physical fitness are known to be closely related [1, 2, 25], and some previous studies have reported that the difference between chronological age and physical fitness age reflects the progression of aging [21, 30]. Therefore, physical fitness is reportedly affected by factors such as lifestyle, physical activity level, and environmental conditions [9, 19], and because an individual’s fitness age is affected not only by genetic factors but also by lifestyle, the level of physical fitness and fitness age differ different depending on the individual’s management method. In this study, according to a multiple linear regression model of health-related fitness indicators, the age determinant of physical fitness in older adults was found to be in a significantly lower-level range. Our study aimed to predict fitness age using only easy-to-measure independent variables, but the adjusted coefficient of determination was considered to be low and insufficient for use in clinical and medical fields. We assumed that if a prediction equation is developed that considers age and physical fitness variables for Korean adults and older adults, the physical fitness age of the Korean adults and older adults can be estimated more accurately. However, few scientifically rigorous studies are available, and systematic large-scale studies on the age-of-fitness estimation formula are limited. Therefore, a large-scale study is needed to expand upon these findings.

To the best of our knowledge, this study is the first large-scale study reported in Korea and is expected to present major trends and standardizations in future health-related fitness age analyses. This study has several limitations. First, as we only accumulated data from a large-scale national study in South Korea (The National Fitness Award), our prediction model may not fit people from other countries. However, this study provided a framework for similar studies in other countries to develop fitness age prediction models using their own data sets. Second, biochemical and psychological parameters other than physical variables were not measured.

## Conclusion

In conclusion, we predicted the fitness age values of each group from these physical fitness indicators. The evaluation of fitness age based on health-related physical fitness indicators can indicate the level of physical aging and provide effective customized exercise programs suitable for physical fitness levels. Furthermore, it can be used as an indicator of longitudinal evaluation of the effectiveness of exercise programs and is expected to be used as a basis for exercise prescription programs along with exercise recommendations at the national level.

## Data Availability

The datasets analyzed in the current study are available from the corresponding author on reasonable request.

## References

[1] Ni W, Yuan X, Zhang Y, Zhang H, Zheng Y, Xu J. Sociodemographic and lifestyle determinants of multimorbidity among community-dwelling older adults: findings from 346,760 SHARE participants. BMC Geriatr. 2023;23(1):419. 37430183 10.1186/s12877-023-04128-1 PMC10331968

[2] Leslie E, Luna V, Gibson AL. Older adult aerobic capacity, muscular strength, fitness and body composition after 20 + years of Exercise Training: a systematic review and Meta-analysis. Int J Exerc Sci. 2023;16(3):620–37. 37622038 10.70252/HSVD9987 PMC10446954

[3] Mann JFE, Chang TI, Cushman WC, Furth SL, Ix JH, Hou FF, Knoll GA, Muntner P, Pecoits-Filho R, Sarnak MJ, et al. Commentary on the KDIGO 2021 Clinical Practice Guideline for the management of blood pressure in CKD. Curr Cardiol Rep. 2021;23(9):132. 34398316 10.1007/s11886-021-01559-3 PMC8366157

[4] Jolliffe DA, Camargo CA Jr., Sluyter JD, Aglipay M, Aloia JF, Ganmaa D, Bergman P, Bischoff-Ferrari HA, Borzutzky A, Damsgaard CT, et al. Vitamin D supplementation to prevent acute respiratory infections: a systematic review and meta-analysis of aggregate data from randomised controlled trials. Lancet Diabetes Endocrinol. 2021;9(5):276–92. 33798465 10.1016/S2213-8587(21)00051-6

[5] Kalache A, de Hoogh AI, Howlett SE, Kennedy B, Eggersdorfer M, Marsman DS, Shao A, Griffiths JC. Nutrition interventions for healthy ageing across the lifespan: a conference report. *Eur J Nutr* 2019, 58(Suppl 1):1–11. 10.1007/s00394-019-02027-z PMC6611748 31254092

[6] Nakagaichi M, Anan Y, Hikiji Y, Uratani S. Developing an assessment based on physical fitness age to evaluate motor function in frail and healthy elderly women. Clin Interv Aging. 2018;13:179–84. 29416326 10.2147/CIA.S146996 PMC5790071

[7] Latorre-Rojas EJ, Prat-Subirana JA, Peirau-Teres X, Mas-Alos S, Beltran-Garrido JV, Planas-Anzano A. Determination of functional fitness age in women aged 50 and older. J Sport Health Sci. 2019;8(3):267–72. 31193284 10.1016/j.jshs.2017.01.010 PMC6523037

[8] Jia L, Zhang W, Chen X. Common methods of biological age estimation. Clin Interv Aging. 2017;12:759–72. 28546743 10.2147/CIA.S134921 PMC5436771

[9] Li Z, Zhang W, Duan Y, Niu Y, Chen Y, Liu X, Dong Z, Zheng Y, Chen X, Feng Z, et al. Progress in biological age research. Front Public Health. 2023;11:1074274. 37124811 10.3389/fpubh.2023.1074274 PMC10130645

[10] Dubina TL, Dyundikova VA, Zhuk EV. Biological age and its estimation. II. Assessment of biological age of albino rats by multiple regression analysis. Exp Gerontol. 1983;18(1):5–18. 6873212 10.1016/0531-5565(83)90046-3

[11] Dyundikova VA, Silvon ZK, Dubina TL. Biological age and its estimation. I. studies of some physiological parameters in albino rats and their validity as biological age tests. Exp Gerontol. 1981;16(1):13–24. 7215475 10.1016/0531-5565(81)90003-6

[12] Li Z, Zhang W, Duan Y, Niu Y, He Y, Chen Y, Liu X, Dong Z, Zheng Y, Chen X, et al. Biological age models based on a healthy Han Chinese population. Arch Gerontol Geriatr. 2023;107:104905. 36542874 10.1016/j.archger.2022.104905

[13] Kimura M, Mizuta C, Yamada Y, Okayama Y, Nakamura E. Constructing an index of physical fitness age for Japanese elderly based on 7-year longitudinal data: sex differences in estimated physical fitness age. Age (Dordr). 2012;34(1):203–14. 21424789 10.1007/s11357-011-9225-5 PMC3260370

[14] Chen PH, Chen W, Wang CW, Yang HF, Huang WT, Huang HC, Chou CY. Association of Physical Fitness Performance tests and anthropometric indices in Taiwanese adults. Front Physiol. 2020;11:583692. 33329032 10.3389/fphys.2020.583692 PMC7718011

[15] Mier CM. Accuracy and feasibility of video analysis for assessing hamstring flexibility and validity of the sit-and-reach test. Res Q Exerc Sport. 2011;82(4):617–23. 22276403 10.1080/02701367.2011.10599798

[16] Beltz NM, Gibson AL, Janot JM, Kravitz L, Mermier CM, Dalleck LC. Graded Exercise Testing Protocols for the Determination of VO(2)max: Historical Perspectives, Progress, and Future Considerations. *J Sports Med (Hindawi Publ Corp)* 2016, 2016:3968393. 10.1155/2016/3968393 PMC5221270 28116349

[17] Park J, Cho B, Kwon H, Lee C. Developing a biological age assessment equation using principal component analysis and clinical biomarkers of aging in Korean men. Arch Gerontol Geriatr. 2009;49(1):7–12. 18597867 10.1016/j.archger.2008.04.003

[18] Jee H, Jeon BH, Kim YH, Kim HK, Choe J, Park J, Jin Y. Development and application of biological age prediction models with physical fitness and physiological components in Korean adults. Gerontology. 2012;58(4):344–53. 22433233 10.1159/000335738

[19] Toyoshima K, Seino S, Tamura Y, Ishikawa J, Chiba Y, Ishizaki T, Fujiwara Y, Shinkai S, Kitamura A, Araki A. Difference between physical fitness age based on physical function and chronological age is Associated with obesity, hyperglycemia, depressive symptoms, and low serum albumin. J Nutr Health Aging. 2022;26(5):501–9.35587763 10.1007/s12603-022-1786-8

[20] Husted KLS, Brink-Kjaer A, Fogelstrom M, Hulst P, Bleibach A, Henneberg KA, Sorensen HBD, Dela F, Jacobsen JCB, Helge JW. A model for estimating Biological Age from physiological biomarkers of healthy Aging: cross-sectional study. JMIR Aging. 2022;5(2):e35696. 35536617 10.2196/35696 PMC9131142

[21] Beltran-Sanchez H, Palloni A, Huangfu Y, McEniry MC. Modeling biological age and its link with the aging process. PNAS Nexus. 2022;1(3):pgac135. 36741436 10.1093/pnasnexus/pgac135 PMC9896935

[22] Salih A, Nichols T, Szabo L, Petersen SE, Raisi-Estabragh Z. Conceptual overview of Biological Age Estimation. Aging Dis. 2023;14(3):583–8. 37191413 10.14336/AD.2022.1107 PMC10187689

[23] Kim SW, Park HY, Jung H, Lee J, Lim K. Estimation of Health-related physical fitness using multiple Linear regression in Korean adults: National Fitness Award 2015–2019. Front Physiol. 2021;12:668055. 34054580 10.3389/fphys.2021.668055 PMC8155701

[24] Kim SW, Park HY, Jung H, Lim K. Development of functional fitness prediction equation in Korean older adults: the National Fitness Award 2015–2019. Front Physiol. 2022;13:896093. 35620610 10.3389/fphys.2022.896093 PMC9127254

[25] Yoon DH, Lee JY, Song W. Effects of Resistance Exercise training on cognitive function and physical performance in cognitive Frailty: a Randomized Controlled Trial. J Nutr Health Aging. 2018;22(8):944–51.30272098 10.1007/s12603-018-1090-9

[26] Park HY, Jung WS, Hwang H, Kim SW, Kim J, Lim K. Predicting the resting metabolic rate of young and middle-aged healthy Korean adults: a preliminary study. Phys Act Nutr. 2020;24(1):9–13. 32408408 10.20463/pan.2020.0002 PMC7451844

[27] Nakamura E, Moritani T, Kanetaka A. Biological age versus physical fitness age. Eur J Appl Physiol Occup Physiol. 1989;58(7):778–85. 2737197 10.1007/BF00637391

[28] Nakamura E, Moritani T, Kanetaka A. Further evaluation of physical fitness age versus physiological age in women. Eur J Appl Physiol Occup Physiol. 1998;78(3):195–200. 9720996 10.1007/s004210050407

[29] Jeong D, Park S, Kim H, Kwon O. Association of Carotenoids Concentration in blood with physical performance in Korean adolescents: the 2018 National Fitness Award Project. Nutrients 2020, 12(6). 10.3390/nu12061821 PMC7353445 32570892

[30] Chen M, Yerramalla MS, van Hees VT, Bloomberg M, Landre B, Fayosse A, Benadjaoud MA, Sabia S. Individual barriers to an active lifestyle at older ages among Whitehall II study participants after 20 years of follow-up. JAMA Netw Open. 2022;5(4):e226379. 35389501 10.1001/jamanetworkopen.2022.6379 PMC8990327

